# Polyurethane Adhesives for Wood Based on a Simple Mixture of Castor Oil and Crude Glycerin

**DOI:** 10.3390/ma16237251

**Published:** 2023-11-21

**Authors:** Tábata Larissa Corrêa Peres, Felipe Vahl Ribeiro, Arthur Behenck Aramburu, Kelvin Techera Barbosa, Andrey Pereira Acosta, André Luiz Missio, Mahbube Subhani, Rafael de Avila Delucis

**Affiliations:** 1 Postgraduate Program in Materials Science and Engineering, Federal University of Pelotas, Pelotas 96010-150, RS, Brazil; tcorreaperes@gmail.com (T.L.C.P.); andre.missio@ufpel.edu.br (A.L.M.); rafael.delucis@ufpel.edu.br (R.d.A.D.); 2 Engineering Centre, Federal University of Pelotas, Pelotas 96010-610, RS, Brazil; felipe.vs.ribeiro@gmail.com; 3 Postgraduate Program in Mining, Metallurgical and Materials Engineering, Federal University of Rio Grande do Sul, Porto Alegre 91540-000, RS, Brazil; arthuraramburu@gmail.com (A.B.A.); kelvintecherabarbosa@gmail.com (K.T.B.); andrey.acosta@ufrgs.br (A.P.A.); 4 School of Engineering, Deakin University, Melbourne, VIC 3216, Australia

**Keywords:** crude glycerin, wood bonding, bio-polyols, renewable resources

## Abstract

Developing a new type of polyurethane is essential because conventional options often exhibit shortcomings in terms of environmental sustainability, cost-effectiveness, and performance in specialized applications. A novel polyurethane adhesive derived from a simple mixture of castor oil (CO) and crude glycerin (CG) holds promise as it reduces reliance on fossil fuels and harnesses renewable resources, making it environmentally friendly. Simple CO/CG mixtures, adjusted at three different weight fractions, were used as bio-based polyester polyols to produce polyurethane adhesive for wood bonding. The resulting products are yellowish liquids with moderate-to-high viscosity, measuring 19,800–21,000 cP at 25 °C. The chemical structure of the polyester polyols was characterized using infrared spectroscopy (FTIR), thermogravimetry (TG), and differential scanning calorimetry (DSC). These polyols reacted with polymeric 4,4-methylene diphenyl diisocyanate (p-MDI) at a consistent isocyanate index of 1.3, resulting in the formation of polyurethane adhesives. Crucially, all final adhesives met the adhesive strength requirements specified by ASTM D-5751 standards, underscoring their suitability for wood bonding applications. The addition of CG enhanced the surface and volumetric hydrophobicity of the cured adhesives, resulting in adhesive properties that are not only stronger but also more weather-resistant. Although the thermal stability of the adhesives decreased with the inclusion of CG, FTIR analysis confirmed proper polyurethane polymer formation. The adhesive adjusted for a 2:1 CO:CG weight ratio promoted wood–wood bonding with the highest shear strength, likely due to a higher formation of urethane linkages between hydroxyl groups from the blend of polyols and isocyanate groups from the p-MDI.

## 1. Introduction

Polyurethanes have been used as coatings, cast elastomers, thermoplastic elastomers, rigid foams, semi-rigid foams, sealants, flexible foams, and adhesives, among others [1]. Polyol is the main PU component, as it makes up 60–70% of its weight [2]. Generally, polyether polyols derived from petroleum are employed. In order to reduce the reliance on fossil-fuel-based products, the use of polyethylene polyols derived from vegetable oils has been investigated in recent years [3,4]. These studies are motivated by the economic, environmental, and social advantages associated with the use of commercially disregarded oils to produce high-value products [5]. Also, insights from recent works in the field of bio-based polyurethanes, including the study by Kaur et al. [6], emphasize the significance of exploring renewable sources for polyol production. Some options for vegetable oils that can be used as polyols include soybean, sunflower, palm, rapeseed, cottonseed, and linseed oil. Some of these oils require a prior chemical reaction, while others can be directly applied because they are naturally rich in hydroxyl groups.

Castor oil (CO) (*Ricinus communis*) has an average hydroxyl functionality of 2.7, with 70% of these hydroxyls coming from triols (such as glycerol triricinoleate) and 30% from diols (such as triacylglycerols), and thus, this oil does not contain any monohydric alcohols [7]. Due to its natural hydroxyl group content and wide availability, CO is found to be a versatile and valuable raw material for direct use in the PU industry. However, the hydroxyl content of CO (equal to 160 mg KOH/g of oil) is considered low for many PU applications (such as adhesives and rigid foams) [8]. Therefore, glycerol (C_3_H_8_O_3_) was implemented in some studies to increase the hydroxyl content of CO [9,10]. Other strategies were explained by Malik and Kaur [11] and Aggarwal et al. [12].

In recent years, there has been a growing interest in processing crude glycerol, which is a byproduct of the biodiesel industry and can be used to generate other value-added products, such as fine chemicals and polymers. Research efforts on new applications for CG can contribute towards reducing environmental impacts associated with its production and disposal. The chemical composition of a typical CG involves glycerol, water, and other components, such as free fatty acids, methanol, and salts. Previous studies have reported successful production of glycerol-based polyols with suitable properties for PU applications [13,14,15,16]. Although some impurities present in crude glycerol, such as fatty acids and methyl esters of fatty acids, may impair the performance of certain PU-derived products [15], this material is considered promising for the partial or even total replacement of some vegetable and mineral oils, due to its low cost and the fact that it does not stress the food chain [8].

Current research in the wood industry is prominently focused on the development of environmentally friendly adhesives [17]. These adhesives play a vital role in a variety of engineered wood products and can be categorized by their solvent type (such as low-boiling-point solvents, water-based solvents, or solvent-free), adhesive type (including hot-melt, thermosetting, or contact adhesives), physical form (ranging from pastes and liquids to pellets), and chemical composition (epoxies, urea-formaldehyde, or polyurethanes) [18]. The performance of PU adhesives for wood bonding hinges on a multitude of factors, encompassing substrate surface smoothness, pH levels, the presence of extractives, and the amount of cutting residues. A primary determinant of adhesive performance is the hydroxyl content of the adhesive [19]. Moreover, assessing the durability of wood adhesives is critical to ensuring the reliability and longevity of wood-based products and structures. Comprehensive evaluation necessitates subjecting these adhesives to conditions that replicate real-world scenarios, including exposure to moisture, temperature fluctuations, and challenging acidic or alkaline environments. These conditions mirror the challenges wood-adhesive bonds may face during their service life [20,21].

Adhesives commonly used for wood bonding, such as urea-formaldehyde or phenol-formaldehyde, are recognized for their high shear strength [18]. However, these adhesives can be susceptible to hydrolysis in certain cases, potentially affecting their long-term performance [18]. Furthermore, these traditional adhesives raise health concerns and contribute to air pollution due to the emission of formaldehyde gas [19]. In fact, in 2004, the International Agency for Research on Cancer reclassified formaldehyde as “carcinogenic to humans” [22]. Currently, formaldehyde is categorized as a carcinogenic and toxic substance, with an acute oral toxicity of 100 mg/kg for rats [23]. In contrast, polymeric diphenylmethane diisocyanate (p-MDI), a common component in PU adhesives, is notably less toxic than formaldehyde, with an acute oral toxicity exceeding 2000 mg/kg in rats [23]. Polyurethane (PU), as an alternative, offers several advantages for wood bonding. It exhibits good surface wetting, non-toxicity after complete curing, and versatile formulation [24]. PU also interacts effectively with substrates through polar interactions, such as hydrogen bonding [25]. Its relatively low molecular weight allows for penetration into porous substrates and the formation of covalent bonds with active hydrogen atoms on the substrate’s surface [24]. These characteristics result in versatile performance, strong adhesion, suitability for low-temperature applications, thermal stability, rapid curing, high toughness, and good chemical resistance [24]. PU has a proven track record for bonding wood [26]. In fact, research has indicated that PU adhesives produced with glycerol-based polyols outperform some commercial PU adhesives designed for wood bonding, particularly in terms of shear strength at the bond line [8]. Additionally, the authors have suggested that the addition of glycerol is positive for accelerating the curing of the adhesives, which can be beneficial for the production of fast-curing adhesives [8].

In recent times, the realm of scientific inquiry has witnessed significant strides in the quest to develop polyurethane foams using unconventional combinations of CO and CG. Researchers, such as Hejna et al. [13], embarked on the formulation of bio-based polyols hinged upon the utilization of CG and CO. These biopolyols, integrated at variable substitution rates (ranging from 0 to 70 wt.%), were synthesized through the polymerization of crude glycerol under vacuum conditions at 180 °C for 6 h. The incorporation of bio-polyols led to a notable reduction in average cell size by up to 25%, a substantial increment in closed cell content by nearly 2%, and a decrease in the thermal conductivity coefficient from 23.4 to 21.8 mW/m K.

In a parallel endeavor, Gurgel et al. [27] ventured into the production of flexible polyurethane foams using eucalyptus-based black liquor, CO, and crude glycerol. Their approach involved the glycerolysis of black liquor and crude glycerol in a hot bath maintained at 110 °C with constant stirring at 500 rpm for 4 h. Subsequent steps included the addition of CO under modified atmosphere conditions (N_2_) at 225 °C to prevent oxidation reactions, followed by magnetic stirring at 500 rpm for 120 min. The outcome was the successful generation of flexible polyols for the production of polyurethane foams.

On the other hand, the utilization of biomass without the need for extensive pretreatment has captured the attention of industry stakeholders due to its potential for streamlining production processes, reducing costs, and expediting overall production [28]. Carriço et al. [29], in their pursuit of optimal formulations, experimented with various proportions of crude glycerol and CO. Through compression parallel to the rise direction and thermal conductivity assessments, they identified that the most promising formulation comprised 10% crude glycerol and 90% CO (*w*/*w*, molar ratio = 1:1).

Delucis et al. [2] embarked on the preparation of polyols for rigid polyurethane foams by mechanically homogenizing mixtures of castor oil (CO), crude glycerin (CG), and varying NCO/OH ratios. Their efforts resulted in the successful production of semi-rigid and rigid biobased PU foams, with an NCO/OH ratio of 1.2 yielding rigid foams with superior mechanical properties. Meanwhile, Carriço et al. [28] also explored the mechanical homogenization of physical mixtures, incorporating Eucalyptus Kraft pulp lignin, CO, and CG in different proportions. Their research highlighted the significant impact of CO and the NCO/OH molar ratio on enhancing urethane cross-linking, ultimately leading to increased stiffness in the resulting foams.

These endeavors illuminate the potential of unconventional polyols derived from CO and CG in the realm of polyurethane foam production. As we delve into the depths of this research, it becomes increasingly evident that when a polyol is aptly tailored for the rigors of rigid polyurethane foam production, it signifies a promising avenue for the formulation of polyurethane adhesives for wood. The underlying principle lies in the consistent compatibility and reactivity of polyols with isocyanates across diverse applications, underscoring the transformative possibilities this burgeoning field offers. Considering that the hydroxyl content of glycerol is known to be between 250 and 350 mg KOH/g of oil [9], the present study aims to investigate the influence of variable percentages of CG in CO for producing PU adhesives for wood bonding.

## 2. Materials and Methods

### 2.1. Preparation and Characterization of the Polyols

Mixtures with weight ratios of 2:1, 3:1, and 4:1 for CO (acquired from Dinâmica company, located in Indaiatuba, Brazil) and CG (donated by OleoPlan company, located in Veranópolis, Brazil) were used as plant-based polyols. These polyols were prepared by simple mixing of the CO and CG on a mechanical stirrer set at a speed of 1000 rpm for 2 h.

The polyols were characterized for hydroxyl content, viscosity, apparent density, moisture content, and colorimetry. The hydroxyl content was determined according to the rule of mixtures based on known values for these materials (160 and 1800 mg KOH/g for CO and glycerol, respectively) and also considering the composition of the CG reported by the supplier. According to this company, the studied CG is composed of glycerol (80%), ash (8%), and moisture (12%). The moisture content of the polyols was defined using the same criterion.

The viscosity of the polyols was determined using a Brookfield DV II+ viscometer, equipped with coaxial cylindrical shafts SC4–21, sample cups, and a water circulation jacket. The sample (10 mL) was transferred to the viscometer’s cylindrical cell and was evaluated at 20 °C using a n° 21 spindle and a shear rate of 50 s^−1^. The apparent density of the polyols was measured using a graduated pipette and an analytical balance. The colorimetry was performed visually, comparing the coloration of each blend of polyols with the Gardner scale [30].

### 2.2. Preparation and Characterization of the Adhesives

The diphenylmethane diisocyanate (p-MDI) (acquired from Polisystem company, located in Porto Alegre, Brazil) was used as the NCO source. A constant NCO/OH ratio of 1.3 was applied for all adhesives. These adhesives were prepared by simple mixing of the CO and CG on a mechanical stirrer set at a speed of 1000 rpm for 60 s. It is pertinent to mention that a phase separation process occurred once the mixture was kept undisturbed for some minutes.

Gel time, surface drying time, and tack-free time were determined according to ASTM D7487 [31] in triplicate. The gel time was determined based on the period between the start of mixing the components and the moment when there was a significant change in the consistency of the mixture. To determine the surface drying time, a wooden rod was inserted successively until a visible mark appeared. The tack-free time was determined based on the period between the start of mixing the components and the moment when the same wooden stick no longer adhered to the adhesive.

Chemical characteristics were evaluated by Fourier transform infrared spectroscopy (FTIR) coupled with an attenuated total reflection (ATR) device in a Perkin Elmer Spectrum (model 400) equipment. The reported spectrum of each sample is the average of 32 scans within the range of 600–4000 cm^−1^ at a scanning interval of 4 cm^−1^. Thermal stability testing was conducted using a Q50 thermogravimetric (TG) analyzer from TA Instruments. The analysis was carried out in a nitrogen atmosphere, with a heating rate of 10 °C per minute. The temperature range for the analysis spanned from room temperature, approximately 20 °C, to 800 °C. Differential scanning calorimetry (DSC) analysis was conducted using a Q20 DSC equipment from TA Instruments. The procedure involved the use of a 5 mg sample in a nitrogen atmosphere with a flow rate of 50 mL/min. The sample was initially heated from 25 °C to 250 °C to investigate its thermal transitions. Following the first heating, the sample was subsequently cooled and then subjected to a second heating, both at a rate of 10 °C per minute in ramp mode. This sequence of heating-cooling-heating is a common practice in DSC analyses to assess the thermal behavior of materials. The measurements of apparent static contact angle were carried out in triplicate with a Kruss goniometer (model DSA 100 S). In this experiment, we applied a 15 μL deionized water droplet onto each adhesive formulation. Subsequently, we recorded the contact angles six times, starting from 0 s and up to 60 s. Regarding water absorption of the adhesives, 5 prismatic samples with a dimension of 2 × 2 × 2 cm were immersed in distilled water in a 2 L beaker and weighed every 25 h using an analytical scale.

### 2.3. Bond Properties at the Wood-to-Wood Interface for Adhesive Evaluation

**Figure 1:**
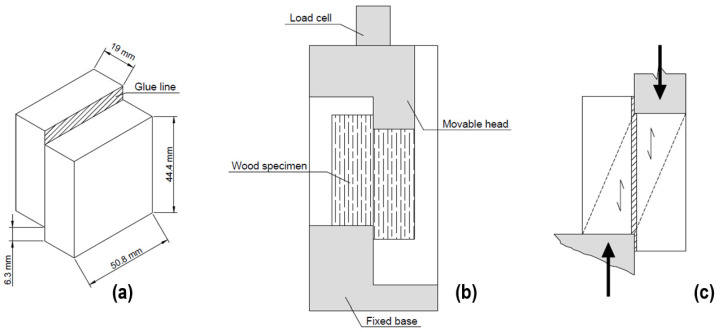
Schematic representation of adhesive bond line shear test components. Where: 3D view of the specimen (**a**), position of the specimen in the equipment (**b**) and shearing of the specimen (**c**).

**Figure 2:**
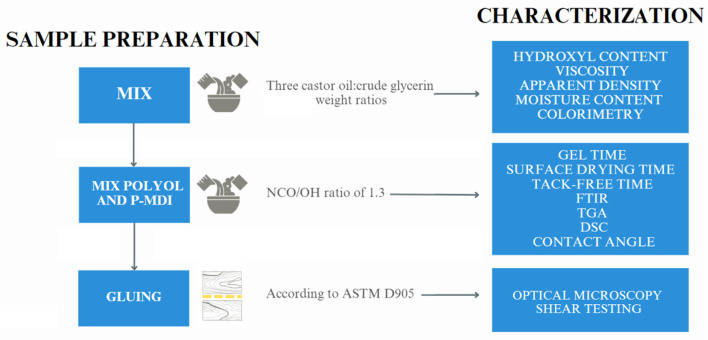
Sequential sample preparation and characterization testing flowchart.

The adhesives were applied to pine (*Pinus elliottii*) woods that came from an approximately 15-year-old planted forest located at Piratini, Brazil. Based on background studies from the research group using wood of the same species and origin, the mechanical properties of this material are as follows: 11–15 MPa for compressive modulus, 39–43 MPa for compressive strength, 7–11 GPa for bending modulus, 62–76 MPa for bending strength, and 31–44 MPa for surface hardness [32,33]. Prior to that, the samples were prepared with sandpaper number 60, according to ASTM D905 [34]. These pieces had dimensions of 5.10 × 5.40 × 2.00 cm (tangential × longitudinal × radial). The adhesive was applied on the surfaces of the boards which would be bonded together at an amount of 200 g/m^2^. The boards were then cold pressed at a pressure of ~0.032 MPa for 24 h using a hydraulic press. After bonding, the lateral plane of the glue line was evaluated using an optical microscope. To evaluate the influence of weathering on adhesion efficiency, samples were subjected to various environmental conditions, as shown in Table 1. These weathering tests were conducted following the procedures outlined in previous studies [20,21]. After that, shear tests due to compressive forces were carried out at a speed of 5 mm/min according to ASTM D905 using an Emic universal testing machine (model DL 30000, EMIC, Tokyo, Japan). A total of ten samples per group were tested. Figure 1 illustrates the key components of the adhesive bond line shear test setup, including the sample configuration (A), loading arrangement (B), and representation of the applied shear stress (C). This schematic provides an overview of how these elements are integrated into the testing procedure. Furthermore, Figure 2 presents a visual representation of the sequential sample preparation and characterization testing process outlined in this study.

## 3. Results and Discussion

### 3.1. Physical and Chemical Characteristics of Polyols

Data regarding hydroxyl content, viscosity, apparent density, moisture content, and color of the polyols are described in Table 2. The hydroxyl content of the 2:1 polyol was found to be 21.25%. The hydroxyl amount decreases with the increasing CO content. The hydroxyl amount decreased by 18% for a 3:1 ratio, whereas for a 4:1 ratio, the reduction amount was 29%. This is probably due to the lower amount of CO in the latter two cases. This phenomenon occurs because glycerol is a triol that, in addition to three carbon atoms and eight hydrogen atoms, has three hydroxyl groups (-OH) per molecule [28]. This chemical characteristic also confers high miscibility in water and allows glycerol to be easily oxidized to aldehydes or carboxylic acids.

The same behavior was observed for viscosity. Although the viscosity of both oils can be considered high, the higher hydroxyl content and the presence of inorganic impurities were responsible for an increase in the viscosity of the CG. These viscosity levels are similar to those obtained by some authors [8,35], and higher than other ones [36,37,38] while producing PU using polyols. In order to adjust the viscosity levels, it is possible to add acetyl acetate to the polyol, which should result in a significant reduction of these levels [39].

A regular density pattern was observed among the polyol proportions. Additionally, the moisture content of the polyols was below 1% in all cases. According to Vale et al. [40], beyond this level (above 1% moisture content), cross-linking formation becomes accelerated, which is detrimental in PU adhesives for wood bonding. Furthermore, when the polyol contains high moisture content, it can negatively affect the chemical reactivity of the hydroxyl groups, leading to a reduction in adhesion effectiveness. On the contrary, if the polyol moisture content is too low, it may result in an incomplete reaction between the polyol and p-MDI, which can also lead to poor adhesion [41,42].

The colors of all the polyols turned yellow, which is typical for CO derivatives. Therefore, the higher the content of CO, the more yellow the polyol was. CO is widely described in the literature as a colorless or pale-yellow liquid [43], while CG is commonly referred to as a dark brown liquid [44]. This elucidates the results presented herein, which have been reported in Table 2 according to the Gardner scale.

### 3.2. Adhesive Polymerization and Viscosity

When comparing the curing times of the adhesives, the 2:1 CO:CG ratio yielded slightly lower values compared to the same associated with the 3:1 and 4:1 ratios (Table 3). This indicates that CG accelerated the reactions between the polyols and the p-MDI. The gel time of the adhesives was found to be inversely proportional to the hydroxyl content, which is associated with the greater difficulty for p-MDI reacting with hydroxyls from the CG [45]. These observed differences in reaction times also suggest that the addition of CG led to a higher cross-linking rate between p-MDI and polyol, which was observed in a previous study for polyols applied in rigid PU foams [9].

The gel time in PU adhesives depends on factors such as the type of curing agent used, environmental temperature, and the amount of catalyst added [46]. Increases in gel time have important implications in the manufacturing of PU products, as it affects the available time for molding or other fabrication processes before the resin starts to solidify. In contrast, increases in gel time allow for a longer working time for PU adhesives before bonding begins to occur, which can be advantageous in some applications. The gel time, surface drying time, and free adhesion time were very similar when comparing the 3:1 and 4:1 CO:CG ratios, indicating that variations in the CO:CG ratio tend to converge or reach the optimum amount in terms of curing times beyond the 2:1 ratio.

It is known that the adhesion strength varies considerably according to the wettability and chemical nature of both the adhesive and substrate. The wettability of a particular surface is a measure of its attractiveness to a liquid (e.g., a CO-based PU adhesive), and is affected by the chemical composition, surface energy, and texture of the surface. An ideal adhesive should spread easily and achieve good molecular contact with the surface, which occurs more efficiently when the adhesive and substrate have similar polarities [47].

**Figure 3:**
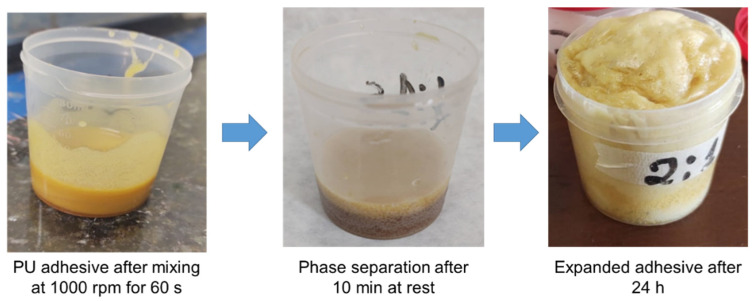
Expansion of the developed PU adhesive as a function of time.

A process of decantation (phase separation) took place after 10 min of resting the adhesive in an open plastic container (Figure 3). After a few hours, it was also observed that the adhesive began to expand slowly, and this expansion was considered completed on the following day (approximately 24 h later). According to Aristri et al. [23], the expansion of PU adhesives during curing can be positive for two reasons: (i) this expansion can help fill gaps or voids in the bond line between the wood pieces; (ii) this expansion can help reduce the slippage between the wood pieces during the adhesive curing, improving the alignment precision and avoiding misalignments.

### 3.3. Chemical and Physical Properties of Adhesives

**Figure 4:**
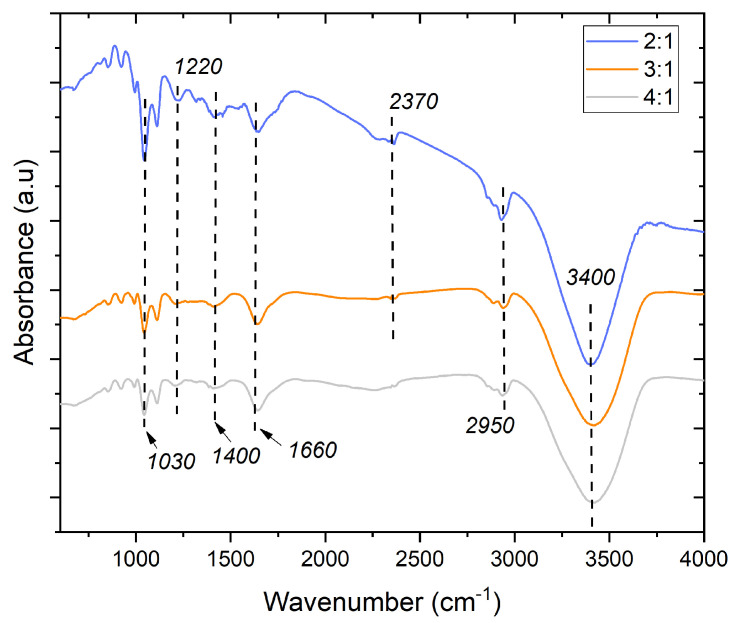
FTIR spectra of the cured adhesives, where 2:1, 3:1, and 4:1 represent the studied CO:CG ratios.

The FTIR spectra of the cured adhesives are shown in Figure 4. The peak at 1030 cm^−1^ is related to the C-H bonding vibrations, while the prominent bands at 1220 cm^−1^ and 1660 cm^−1^ are attributed to the asymmetric stretching vibrations of the C-O bonds and the C=O stretching vibrations of the urethane groups, respectively, and the peak at 1400 cm^−1^ is attributed to the C-H bonding vibrations [38,48,49]. The coexistence of both 1030 cm^−1^ and 1400 cm^−1^ peaks indicates the presence of multiple types of C-H bonds in the adhesive, each contributing to its molecular structure. Separately, the bands between 2850 and 2950 cm^−1^ correspond to C-H stretching vibrations and the band in the region of 3400 cm^−1^ is attributed to OH groups [2,37,50]. These bands indicate proper polymer formation regardless of the studied formulation. From a practical standpoint, these observations indicate that the CO:CG ratio can significantly impact the degree of chemical reaction and cross-linking within the adhesive. The detection of unreacted NCO groups in the 2:1 and 3:1 adhesives with higher CG content suggests that achieving complete chemical conversion may be more challenging under these conditions.

The peak around 2370 cm^−1^, related to the presence of unreacted NCO groups [45,46,47,48,49,50,51,52,53], was only detected in the 2:1 and 3:1 adhesives, where the content of CG was higher. This again indicates that the addition of CG hindered the consumption of hydroxyls by the NCO groups of p-MDI, even considering that a stoichiometric NCO/OH ratio of 1.3 was maintained in all cases. Therefore, adjusting the adhesive formulation and reaction conditions may be necessary to ensure complete chemical conversion and achieve the desired adhesive characteristics.

**Figure 5:**
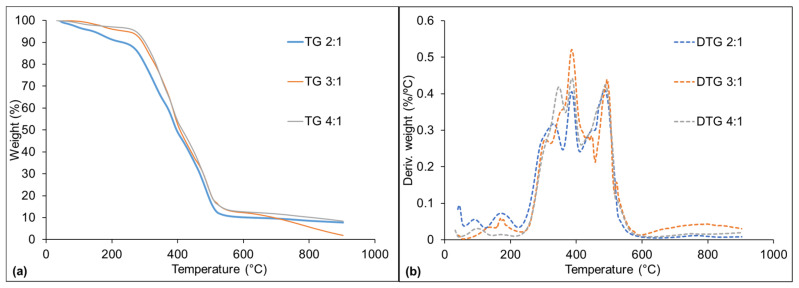
Thermogravimetric curves (**a**) and their derivative (**b**) curves of the cured adhesives, where 2:1, 3:1, and 4:1 represent the studied CO:CG ratios.

Figure 5 illustrates the thermal decomposition behavior of the PU adhesives in terms of TG (thermogravimetry) and DTG (derivative thermogravimetry) curves, wherein TG measures changes in mass and DTG provides insights into the rate of mass change at specific temperature points. These curves show that all of the PU adhesives showed similar curve shapes, indicating that the addition of CG in different proportions did not lead to the formation of new chemical structures. The first degradation step occurred at around 100 °C and can be associated with the loss of residual water. The degradation of free NCO groups started at around 180 °C [18]. At this stage, only the 2:1 and 3:1 adhesives showed peaks in the DTG graph, confirming the results obtained by FTIR, which indicated that the 4:1 adhesive had all of its NCO groups consumed. The consequences and practical implications of the behavior where NCO (isocyanate) groups are completely consumed in the context of polyurethane adhesives can vary depending on the intended application and the properties required for that specific use. In general, cross-linking usually leads to stronger adhesive bonds, enhancing the overall strength and durability of the adhesive joint [54]. Also, cross-linked adhesives tend to exhibit improved thermal stability, with a higher heat resistance.

Within the 270–510 °C temperature range, there is another decomposition stage, which can be related to the structural decomposition of organic chains, mainly governed by the cleavage of urea groups and degradation of urethane groups, which preferably begin from side chains [9]. In simpler terms, at temperatures between 270 and 510 °C, the adhesive undergoes a process where its organic chains start to break down. This breakdown is mainly due to the cleavage of certain chemical groups, namely urea and urethane, and these reactions often begin at the branches or side chains of the adhesive’s molecular structure. According to Malik et al. [51], the reactions occurring in this temperature range involve the formation of CO_2_, CO, as well as amines and aldehydes, due to the breaking of double and single bonds, such as C=O, C=C, C-O, and C-H. The last degradation stage (after 530 °C) is attributed to the formation of fixed carbon [18]. These observations shed light on the thermal behavior and chemical transformations of the adhesive, which are essential for selecting suitable adhesives for specific applications, including temperature-sensitive applications, applications where gas emission or chemical stability is a concern, high-temperature applications, and so on.

Among the studied adhesives, the 2:1 one showed the lowest thermal stability throughout the thermogravimetric profile, which indicates that the addition of CG negatively affects this property. According to the literature, there are two probable reasons for this decrease in thermal stability when CG is added: (i) increase in acidity during the esterification reaction of CG with NCO groups, since it can be converted into acids and this higher acidity of the system reduces the thermal stability of the adhesive; (ii) increase in molecular mobility, since CG is a low molecular weight agent that can be easily released from the polymer, leaving behind empty spaces in the polymer matrix through which the polymer molecules can move [8,13,21].

**Figure 6:**
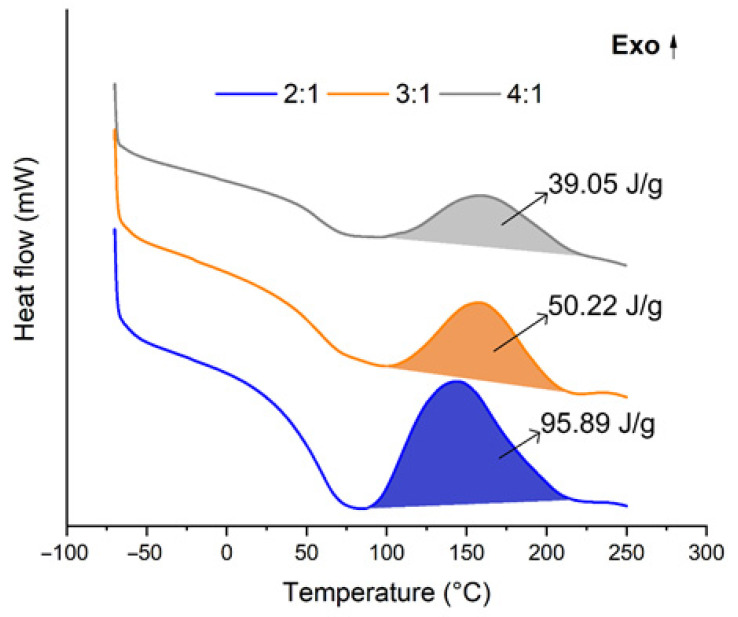
DSC curves of the cured adhesives and enthalpy values (ΔH), where 2:1, 3:1, and 4:1 represent the studied CO:CG ratios.

The DSC (differential scanning calorimetry) curves shown in Figure 6 represent the change in heat flow into or out of the sample relative to a reference material as the temperature is varied. Physically, the DSC curve provides information about the energy changes that occur within a sample as it undergoes phase transitions, chemical reactions, or other thermal processes. Comparing the adhesives in terms of DSC curves, there is similar behavior among them. It is possible to highlight three important regions along the thermograms located approximately at 70 °C, 140 °C, and 170 °C. The first region can be attributed to the dissociation of small unstructured rigid segments [55]. In simpler terms, during this thermal event around 70 °C, certain components or segments within the adhesive may change their structure or arrangement due to the absorption of heat energy. These “small unstructured rigid segments” could be molecular groups or domains that are relatively stable at lower temperatures but become less structured or organized as the temperature increases.

The second and/or third region refers to the degradation of crystalline segments in the polymer backbone. In addition, in all PU adhesives, two transitions can be identified by an exothermic event followed by an endothermic peak at −55 °C and 50 °C. The first transition is due to soft segments of the system containing polyol, while the second transition is due to rigid segments containing urethane linkages [26,51]. Similar results have been obtained for analogous materials [56]. Moreover, the exothermic peak located in the range of 100–200 °C is associated with a release of energy due to curing steps, crystallization, or other irreversible chain rearrangements [26]. The area under the DSC curve is proportional to the enthalpy of thermal transitions, representing the energy released or absorbed during material changes. Considering this relationship, we can presume that a greater number of linkages were formed with the addition of CG. This presumption is supported by the observation that the areas under the curves in Figure 5 increased in the following order: 2:1 > 3:1 > 4:1. In this context, increased cross-linking generally results in enhanced thermal stability. However, the addition of CG reduced this property, which can be attributed to the presence of unreacted isocyanate (NCO) groups.

Moreover, the depth of the valley observed in the temperature range of 80–100 °C differed among the adhesive. The 2:1 mixture, which contained a higher proportion of CG, exhibited a more profound valley. This can be attributed to the presence of impurities in CG, such as fatty acids and methyl esters of fatty acids [8,15] that have distinct thermal transitions in this temperature range, contributing to the increased heat absorption and a deeper valley. Also, the intensity of the peak in the temperature range of 120–160 °C varied, with the 2:1 mixture showing a higher peak intensity. The elevated intensity may be linked to the higher CG content, suggesting potential interactions between CO, CG, or impurities within CG, influencing the thermal behavior of the adhesive.

**Figure 7:**
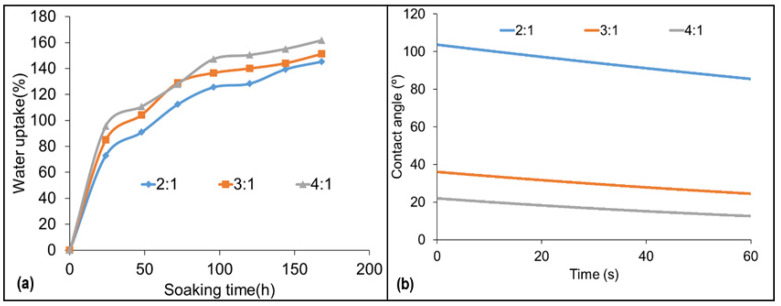
Water uptake (**a**) and contact angle (**b**) of the cured adhesives, where 2:1, 3:1, and 4:1 represent the studied CO:CG ratios.

Figure 7 provides insights into the adhesive’s interaction with water, addressing key properties such as wettability and moisture absorption. Understanding these properties is crucial for selecting adhesives suitable for specific applications, especially those involving exposure to varying environmental conditions. The adhesive produced with the highest CG content (2:1) showed the highest contact angle, indicating a more hydrophobic behavior. This result may be related to the presence of hydrophobic groups in the glycerin structure. Especially in the case of CG, impurities such as free fatty acids, soaps, and other organic compounds may be present. These impurities can react with CO during polyol production and generate products that contain hydrophobic groups, such as alkyls and alkenyls [24]. Separately, the addition of CG, as already mentioned, increases the content of hydroxyl groups in the polyols, which can lead to greater cross-linking of the PU chains during the polymerization reaction. This leads to a denser and less permeable network that contributes to the greater hydrophobicity of these adhesives. This same behavior was reflected in the evolution of water uptake over time, where the 2:1 adhesive absorbed a lower amount of water when compared to the other ratios. In summary, the observed hydrophobic behavior in adhesives, particularly due to the addition of CG and the resulting increase in cross-linking, can lead to improved water resistance and bond performance, making them suitable for applications where exposure to moisture or wet conditions is a concern.

### 3.4. Mechanical Strength of the Wood–Wood Adhesions

**Figure 8:**
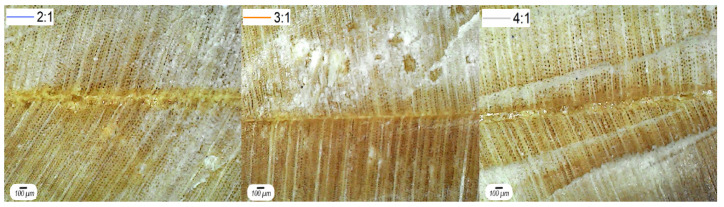
Optical micrographs of the glue lines, where 2:1, 3:1, and 4:1 represent the studied CO:CG ratios.

Figure 8 shows the glue lines for each CO:CG ratio. It can be seen that the 3:1 and 4:1 ratios were equally homogeneous with an approximate thickness of 50 μm. The 2:1 adhesive, on the other hand, presented a thicker glue line (around 70–80 μm), although equally homogeneous. This difference for the 2:1 adhesive is probably due to its higher viscosity, which may hinder wetting and penetration on the wood surface. Regardless of the CO:CG ratio, all failures were cohesive within the adhesive. The observation of cohesive failures within the adhesive indicates that, in most cases, the adhesive itself exhibited good strength, which is a positive aspect for bonded wood products.

The shear strength data showed that the higher the hydroxyl content of the polyol, the stronger the formed glue line (Table 3). Therefore, the 2:1 adhesive stood out, showing the highest levels of shear strength for both control and when subjected to environmental conditions. This result also indicates that, although the glue line formed by wetting the 2:1 adhesive was thicker, indicating a lower spreading rate and anchoring of the adhesive on the wood surface, this polyol originated a better quality adhesion. This result is probably due to the greater formation of urethane bonds between the OH groups of the polyol and the NCO of the p-MDI [21].

According to ASTM D-5751 [57], the average shear strength of a bonded joint should be at least 60% of the value obtained for solid wood testing. Based on the literature, the shear strength levels of Brazilian pine wood vary between 5 and 10 MPa [58]. The adhesives studied here attain a shear strength of 3 to 6 MPa, which meets this standard. It can be seen that the adhesive bonds provided by all adhesives reached this minimum safety value stipulated.

**Figure 9:**
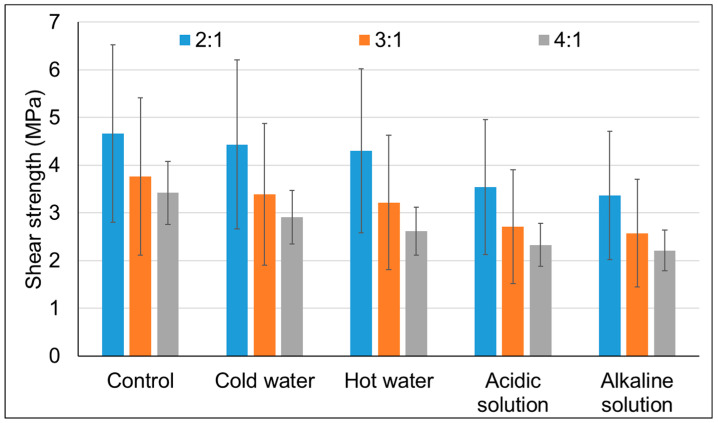
Shear strength of the glue lines. Where 2:1, 3:1, and 4:1 represent the studied CO:CG ratios.

Figure 9 shows the results obtained for the shear strength of the glue lines for different CO:CG ratios after the weathering test.

The 2:1 adhesive showed a good hydrolytic resistance, losing only 5.1% and 7.9% of its strength when exposed to cold and hot water, respectively. The 3:1 and 4:1 ratios showed losses of 10% and 14.58%, and 14.91% and 23.59%, respectively. These results indicate that the higher the content of CG in the polyol, the higher the initial shear strength and the higher the resistance against cold and hot water. It is common knowledge that PU exhibits low resistance under very acidic or very alkaline conditions due to the hydrolysis of esters and urethanes. In the present study, the losses in shear strength were between 20% and 40% in all cases. Again, the 2:1 adhesive outperformed the other ones, followed by the 3:1 adhesive. Finally, it can be said that the shear strengths obtained (close to 5 MPa) are similar to recent analogous studies [21,26], which evaluated wood species including poplar wood, spruce wood, and oak wood. This indicates that the formulations studied are promising for the generation of competitive products in the PU adhesive market for wood. However, it is worth noting that there are reports in the literature of NCO/OH ratios as high as 3 [18,47], which are significantly higher than the 1.3 ratio used in the present study. Implementing higher NCO/OH ratios may further enhance the shear strength values found here.

## 4. Conclusions

This study developed a novel polyurethane adhesive from a simple mixture of castor oil (CO) and crude glycerin (CG). It reduces reliance on fossil fuels but also harnesses renewable resources, making it environmentally friendly. Simple CO/CG mixtures, adjusted at three different weight fractions, were applied as bio-based polyester polyols to produce polyurethane adhesive for wood bonding. The addition of CG increased the hydroxyl content, viscosity, and moisture content, as well as darkened the physical appearance of the polyols. The added CG also reduced the measured reaction times for the 2:1 adhesive compared to the 3:1 and 4:1 adhesives. These adhesives also showed a tendency towards decantation after 10 min and expansion for up to 24 h. FTIR spectra indicated proper PU polymer formation, and the 4:1 adhesive had all NCO groups consumed by the polyol hydroxyls, unlike the adhesives with higher CG contents (i.e., 2:1 and 3:1), which showed unreacted NCO groups, as confirmed by thermogravimetric analysis results. The addition of CG also reduced the thermal stability of the adhesives, although no new chemical group was detected by FTIR, and it also increased the surface and volumetric hydrophobicity of the cured adhesives. Moreover, the higher the addition of CG, the stronger and more resistant against weathering the adhesives were. All final adhesives showed adequate adhesive strength levels according to the ASTM D-5751. Therefore, polyols produced by a simple mixture of CO and CG intended for PU adhesives for wood bonding can be a promising strategy to reach the proper mechanical properties and durability of the adhesives. Future research endeavors may explore the use of these adhesive formulations in diverse applications, such as construction materials, automotive, aerospace, and even medical devices.

## Figures and Tables

**Table 1:** Characterisation of the physical properties of polyols.

Adhesive	Exposure Environment	Exposure Time	N° of Samples
2:1	Ambient water (30 °C)	24 h	10
2:1	Hot water (100 °C)	24 h	10
2:1	Acidic solution H_2_SO_4_ (pH 3 at 70 °C)	1 h	10
2:1	Alkaline solution NaOH (pH 13 at 70 °C)	1 h	10
3:1	Ambient water (30 °C)	24 h	10
3:1	Hot water (100 °C)	24 h	10
3:1	Acidic solution H_2_SO_4_ (pH 3 at 70 °C)	1 h	10
3:1	Alkaline solution NaOH (pH 13 at 70 °C)	1 h	10
4:1	Ambient water (30 °C)	24 h	10
4:1	Hot water (100 °C)	24 h	10
4:1	Acidic solution H_2_SO_4_ (pH 3 at 70 °C)	1 h	10
4:1	Alkaline solution NaOH (pH 13 at 70 °C)	1 h	10

**Table 2:** Physical properties of the polyols.

CO:CG Ratio	2:1	3:1	4:1
Hydroxyl content (mg KOH/g)	582	480	416
Cinematic viscosity (cP)	20,958	20,210	19,817
Apparent density (g/cm^3^)	1.042	1.016	1.019
Moisture content (%)	0.040	0.030	0.024
Color	16	14	5

**Table 3:** Curing times of the adhesives.

CO:CG Ratio	2:1	3:1	4:1
Gel time (min)	8	10	10
Surface drying time (min)	90	110	110
Tack-free time (min)	520	540	540

## Data Availability

Data are contained within the article.
